# Removal of Arsenic (III, V) from aqueous solution by nanoscale zero-valent iron stabilized with starch and carboxymethyl cellulose

**DOI:** 10.1186/2052-336X-12-74

**Published:** 2014-04-24

**Authors:** Mohammad Mosaferi, Sepideh Nemati, Alireza Khataee, Simin Nasseri, Ahmad Asl Hashemi

**Affiliations:** 1 Department of Environmental Health Engineering, Tabriz Health Services Management Research Center, Tabriz University of Medical Sciences, Tabriz, Iran; 2 Center for Water Quality Research (CWQR), Institute for Environmental Research (IER) and department of Environmental Health Engineering, Tehran University of Medical Sciences, Tehran, Iran; 3 Department of Environmental Health Engineering, School of Health, Center of Student Researches, Tabriz University of Medical Sciences, Tabriz, Iran; 4 Research Laboratory of Advanced Water and Wastewater Treatment Processes, Department of Applied Chemistry, Faculty of Chemistry, University of Tabriz, Tabriz, Iran; 5 Department of Environmental Health Engineering, School of Health, Tabriz University of Medical Sciences, Tabriz, Iran

**Keywords:** Metallic iron, Nanoparticles, Water purification, Arsenic, Adsorption

## Abstract

In this work, synthetic nanoscale zerovalent iron (NZVI) stabilized with two polymers, Starch and Carboxymethyl cellulose (CMC) were examined and compared for their ability in removing As (III) and As (V) from aqueous solutions as the most promising iron nanoparticles form for arsenic removal.

Batch operations were conducted with different process parameters such as contact time, nanoparticles concentration, initial arsenic concentration and pH.

Results revealed that starch stabilized particles (S-nZVI) presented an outstanding ability to remove both arsenate and arsenite and displayed ~ 36.5% greater removal for As (V) and 30% for As (III) in comparison with CMC-stabilized nanoparticles (C-nZVI). However, from the particle stabilization viewpoint, there is a clear trade off to choosing the best stabilized nanoparticles form. Removal efficiency was enhanced with increasing the contact time and iron loading but reduced with increasing initial As (III, V) concentrations and pH. Almost complete removal of arsenic (up to 500 μg/L) was achieved in just 5 min when the S-nZVI mass concentration was 0.3 g/L and initial solution pH of 7 ± 0.1. The maximum removal efficiency of both arsenic species was obtained at pH = 5 ± 0.1 and starched nanoparticles was effective in slightly acidic and natural pH values. The adsorption kinetics fitted well with pseudo-second-order model and the adsorption data obeyed the Langmuir equation with a maximum adsorption capacity of 14 mg/g for arsenic (V), and 12.2 mg/g for arsenic (III).

It could be concluded that starch stabilized Fe^0^ nanoparticles showed remarkable potential for As (III, V) removal from aqueous solution e.g. contaminated water.

## Introduction

Arsenic, a notorious poison, is now recognized to be one of the world’s greatest environmental hazards, threatening the lives of several hundred million people [1]. In many areas of the world, biogeochemical processes have resulted in a release of naturally occurring As into groundwater; in some cases, large regions are affected [2]. On the other hand, uncontrolled anthropogenic activities such as (mining, fossil fuels burning, smelting of metal ores, and use of wood preservatives, pesticides and arsenic additives to livestock feed) may also release arsenic directly to the environment [3-5]. As a result exposure to arsenic compounds is a major concern to public health in both developing and developed countries [6] and removal of arsenic from drinking water is a worldwide priority.

Exposure to elevated arsenic levels has been attributed to adverse health related issues such as changes in skin pigmentation, diabetes, lung ailments, and cancers of the kidney and bladder [7]. Due to its significant toxicity, the World Health Organization has established a value of 10 μg As/L as the maximum contaminant level for total As in potable water [8].

In Iran, the occurrence of geogenic arsenic in some of rural areas in Kurdistan province, located in the West of country is responsible for health problems related to chronic As exposure from drinking water [9]. Arsenic contamination of drinking water has also been detected in Hashtrud county, in the Northwest of the country [10] and Kohsorkh area in the Northeast of Iran [11] where arsenic concentration in water is higher than the National Iranian Drinking Water Standard, 10 μg As/L [12]. Therefore, it is an urgent need to provide Arsenic free drinking water in the mentioned areas.

As well known, arsenic exists in the natural environment mainly in the forms of arsenite [As (III)] and arsenate [As (V)]. Arsenite is more mobile and toxic than arsenate and most removal technologies are efficient when the element is presented in the pentavalent state [13,14].

Removal of arsenic contamination from water can be accomplished by a variety of techniques such as coagulation [15,16], adsorption [17,18], ion exchange [19], membrane filtration [20,21] and biological process [22].

A significant problem encountered in the removal of arsenic from groundwater aquifers and municipal water systems is the presence of arsenic in both arsenic states arsenic (III) and arsenic (V). Arsenic (III) compounds are primarily non-ionic whereas arsenic (V) compounds are ionic at natural water pH [23].

In comparison with other removal methods, zero-valent iron (ZVI, Fe^0^) can simultaneously remove As (V) and As (III) without previous oxidative treatment and does not require the use of additional chemical products, since metallic iron is used for the sustained production of colloidal hydrous ferric oxides (HFO) [14]. ZVI reactions are rather slow, but the process can be notably accelerated using iron nanoparticles (NZVI) [24]. Due to the low amount of nano or micro zero-valent iron used, the mass of the sludge produced in the process is significantly low; so, the flocs may be removed by magnetic techniques, often more efficient and faster than centrifugation or filtration [25].

Despite these advantages, Fe^0^ nanoparticles were found to be lack in stability in water and in the absence of an effective stabilizer, aggregate rapidly (in a few minutes), resulting in micro-, millimeter-scale or even larger aggregates [26,27].

Aggregation and sedimentation can significantly alter the mobility of the nanoparticles in aquatic environment and reduce the efficacy of using these nanoparticles for remediation purposes [28,29].

Surface modification of NZVI by polyelectrolytes, polymers and surfactants, which provide steric and electrostatic stabilization against the particle-particle attractive forces, significantly improves its transport in porous media [30].

Among all reported stabilizers, water soluble polysaccharides have been proved to be the best stabilizer due to their low cost and environmental compatibility [31]. So, it seems that green polysaccharides such as starch and Carboxymethyl cellulose (CMC) can be used as effective protecting agents to enhance the reactivity of iron nanoparticles.

Starch is a nontoxic, biodegradable and inexpensive biopolymer. Recent study by Alidokht et al. [32] indicated that application of starch as the stabilization agent prevents the aggregation of Fe^0^ nanoparticles which causes high Cr (VI) removal efficiency in comparison with nonstabilized NZVI.

CMC is a chemical derivative of cellulose and has carboxylate groups in addition to hydroxyls, which may result in strong interaction between CMC and Fe nanoparticles [31]. CMC is also a food-grade ingredient, nontoxic and biodegradable and this is likely due to the presence of highly biodegradable -OH, −CO-, and -COOH groups [33].

Xu and Zhao [34] test the feasibility of using the CMC-stabilized ZVI nanoparticles for in situ reductive immobilization of Cr (VI) in contaminated water and soils and reported these nanoparticles may serve as a highly soil-dispersible and effective agent for immobilization of chromate.

To the best of our knowledge, the improved removal of arsenic using starch and CMC stabilized zerovalent iron nanoparticles has not yet been reported in details. Keeping in view the high toxicity of arsenic and high capability of polymer stabilized NZVI in removal of various pollutants; the present study investigates the performance of these nanoparticles in removing arsenic species from aqueous solutions for the first time. Since the mechanism of arsenic removal using stabilized NZVI remains unclear, the possible interaction between arsenic and two stabilized NZVI was proposed. The specific aims were: (1) synthesis and characterization of stabilized Fe^0^ nanoparticles; (2) comparison removal efficiency of As (III) and As (V) by starch stabilized nZVI (S-nZVI), CMC stabilized nZVI (C-nZVI) and bare nanoparticles; and (3) determining the effects of environmental factors on the removal ability of selected form of nanoparticles.

## Experimental

### Materials

All chemicals were of reagent grade. Ferrous sulfate heptahydrate (FeSO_4_.7H_2_O), Sodium borohydride (NaBH_4_), Sodium arsenite stock solution (NaAsO_2,_ 0.05 mol/L) were purchased from (Merck Co, Germany). As (V) stock solution (100 mg/L) was prepared from Na_2_HAsO_4_.7H_2_O (GIFT Co) and stored at 4°C. Water soluble starch and sodium carboxymethyl cellulose, (CMC 90 k) were obtained from Fluka and Sigma (UK) Co respectively. The chemical reagents were used directly without further purification.

### Synthesis of nanoparticles

Nano-Fe^0^ particles were synthesized using the borohydride method [35]. A key advantage of this method is its simplicity. It can be safely done in most chemistry lab with simple chemical reagents. Conventionally these nanoparticles can be synthesized by both FeSO_4_.7H_2_O and FeCl_3_.6H_2_O but during the reaction with the borohydride solution, these two different aqueous solution salts show significant and stoichiometrical differences in reaction. Borohydride reacts more rapidly with FeSO_4_.7H_2_O than FeCl_3_.6H_2_O, which is important because it tends to be less oxidized in the solution obtained after synthesis and may save time [36]. For this reason FeSO_4_ is better option for NZVI synthesis.

For preparation of starch-stabilized Fe^0^ nanoparticles, first an aqueous solution of 0.14 M FeSO_4_.7H_2_O (100 ml) and 0.2% (w/w) starch as a stabilizer were stirred with an electric rod in a 500 ml three necked round bottom flask for 15 min to enable the formation of dissolved Fe-starch complexes. The mixture was purged with nitrogen gas to remove dissolved oxygen. Then the Fe^+2^ ions were reduced to Fe^0^ by adding 100 ml of 0.5 M sodium borohydride solution drop-wise into the mixture. After adding the whole borohydride solution, the mixture was stirred for another 30 minutes. Ferrous iron was reduced to zerovalent iron by borohydride according to the following reaction [32].

(1) $$\begin{array}{ll} 2{{\mathrm{Fe}}^{2+}}_{\left(\mathrm{aq}\right)} & +\;{\mathrm{BH}}_{4^{-}\left(\mathrm{aq}\right)}+3H_{2}O_{\left(1\right)}\to 2{{\mathrm{Fe}}^{0}}_{\left(s\right)}\\ & +\;H_{2}{{\mathrm{BO}}_{3^{-}}}_{\left(\mathrm{aq}\right)}+4{H^{+}}_{\left(\mathrm{aq}\right)}+2{H_{2}}_{\left(g\right)} \end{array}$$

The product was an aqueous black suspension that iron nanoparticles were separated by centrifuge at 5000 rpm for 5 min and quickly washed three times with absolute ethanol to remove excess borohydrate. Prepared particles were dried under vacuum overnight and then gently crushed into fine powders.

CMC-Stabilized iron nanoparticles were fabricated with the same procedure where 0.2% NaCMC served as stabilizer for ZVI nanoparticles.

Finally nonstabilized iron nanoparticles (Bare NZVI) were prepared without stabilizers loading.

### Characterization of stabilized nanoparticles

In this study, the most promising iron nanoparticles form for arsenic removal was selected for characterization study by X-ray diffraction (XRD) method for crystal structure and composition analysis and Scanning electron microscopy (SEM) technique. XRD is a versatile, non-destructive technique that reveals detailed information about the chemical composition and crystallographic structure of natural and manufactured materials and is based on constructive interference of monochromatic X-rays and a crystalline sample [37].

XRD pattern of starch stabilized nanoparticles was obtained using a Siemens D5000 (Germany) diffractometer by Scanning from 20° to 85° (2θ) with a step time of 0.3 s and a step of 0.02° (2θ) with monochromatic Cu-K_α_ radiation (40KV, 30 mA, λ = 0.15418 nm).

The morphological features and surface characteristics of the starch and CMC stabilized Fe^0^ nanoparticles were obtained from Scanning electron microscope (SEM) (Hitachi S 4160, 15.0 kV, Japan).

### Batch experimental system

Arsenic removal experiments were carried out using glass Erlenmeyer 250 mL flasks containing 150 mL arsenic solution with determinate concentration at room temperature (25 ± 1°C). Arsenic solutions with desired concentration were prepared by dilution of the stock solution immediately before use.

At the first stage of study, in order to compare arsenic removal potential of S-nZVI, C-nZVI and Bare-nZVI, in each set of experiments, 0.045 g freshly papered nanoparticles was kept in contact with 150 mL arsenic solution of 2 mg/L concentration. An attempt was made to compare the efficiencies of arsenic removal without pH control (i.e. at circum-neutral pH). The experiment was performed under oxic conditions, whereas the presence of oxygen would help to remove arsenic through oxygen-induced corrosion products of iron [38].

The flasks were shaken with an orbit incubator shaker (Melrose park, ILL, No3595, USA); operated at 200 rpm for time periods up to 120 min. Parallel experiments were conducted in the absence of nanoparticles but under otherwise identical conditions (blank samples). Blank samples showed no significant changes. At certain reaction time intervals, suspensions were withdrawn from the reactors by 5 ml syringe and centrifuged at 3000 rpm for 5 min and then filtered through 0.22 μm syringe filters for analysis.

After selecting the most effective zero-iron nanoparticles form for arsenic uptake, at the next step of the study, kinetic batch experiments were conducted at different initial concentrations of arsenic in the range of (0.25-10) mg/L and adsorbent dose of 0.3 g/L at the period of 5–150 min (25 ± 1°C, 200 rpm, pH = 7).

Adsorption isotherms were obtained by equilibrating arsenic solutions of different nanoparticles dose (100, 300, 500, 1000) mg/L at pH = 7 with an initial arsenic concentration of 2 mg/L at room temperature.

To study the effect of pH on removal efficiency, pH of the arsenic solution was adjusted to values in the range of (3–11) by adding 0.1 M NaOH (BDH chemicals Co, UK) and/or HCl (37%, Merck Co) through a manual syringe injection.

The effects of adsorbent dosage and initial arsenic concentration were investigated.

All samples were analyzed for residual arsenic concentration using graphite furnace atomic absorption spectrometer (GFAAS, Buck Scientific, Inc. 210VGP model, USA). All experiments were performed at least by duplicate and the averages were reported. The analysis was carried out using calibration curves with correlation coefficients (R^2^) of 0.999. Standards were re-measured during each set of experiments to assess accuracy and stability in measurements and to assurance adequate instrument performance.

## Results and discussion

### Characterization results

**Figure 1:**
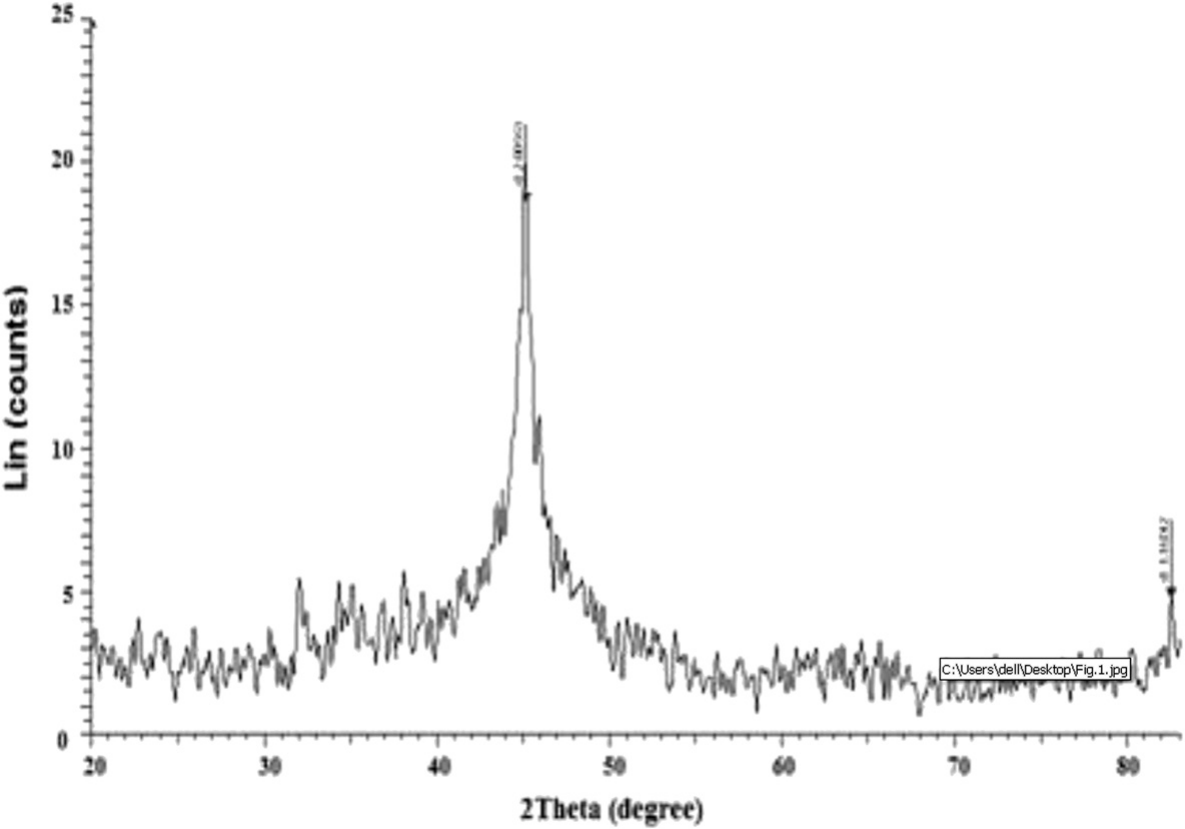
X-ray diffraction spectrum of starch stabilized nanoparticles.

Figure 1 shows the XRD spectra of laboratory prepared Fe^0^ nanoparticles. The characteristic peak at 2θ = 44.7° confirmed the presence of zero-valent iron in freshly prepared S-nZVI. The average crystalline size of the nanoparticles was calculated using Debye Scherrer’s equation from the width at half maximum (β) of the main intense peak (7).

(2) $$D=\frac{k\lambda }{\beta \cos\theta }$$

Where, D is the size of the particles, K the shape factor, about 0.9, λ is the wave-length of the emitted X-rays (0.15418 nm); β is the full width at half maximum of the corresponding peak of the XRD and θ the angle of incidence of X-ray beam. From this analysis it was determined that the synthesized nanomaterials had sizes of 10 nm approximately.

**Figure 2:**
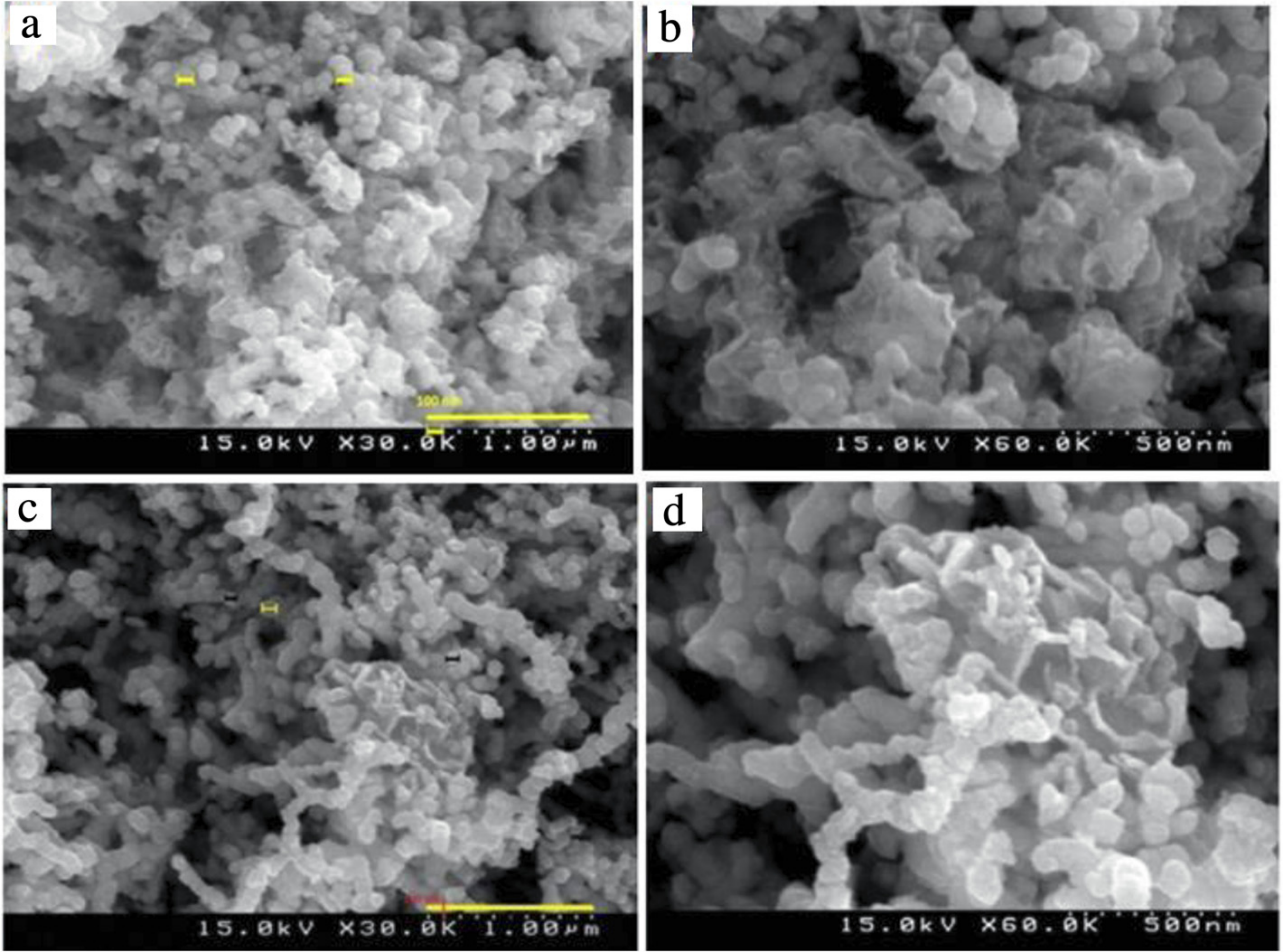
**Characteristic SEM images of stabilized nZVI at different magnifications. (a)** S-nZVI (30000×); **(b)** S-nZVI (60000×); **(c)** C-nZVI (30000×); **(d)** C-nZVI (60000×).

SEM micrographs of S-nZVI at different magnifications are presented in Figure 2a, b. The images reveal that the nanospheral particles of different sizes are not all separated and a fraction of them form nearly dendritic clusters. SEM image of CMC-stabilized iron nanoparticles (Figure 2c, d) is also shown for comparison. It seems that CMC-coated particles are approximately discrete and have a more uniform shape than starch-stabilized particles.

It can be observed that both coated iron nanoparticles have particle size less than 100 nm.

### Selection of most effective Fe^0^ nanoparticles for arsenic removal

#### Comparison of arsenic removal efficiency

**Figure 3:**
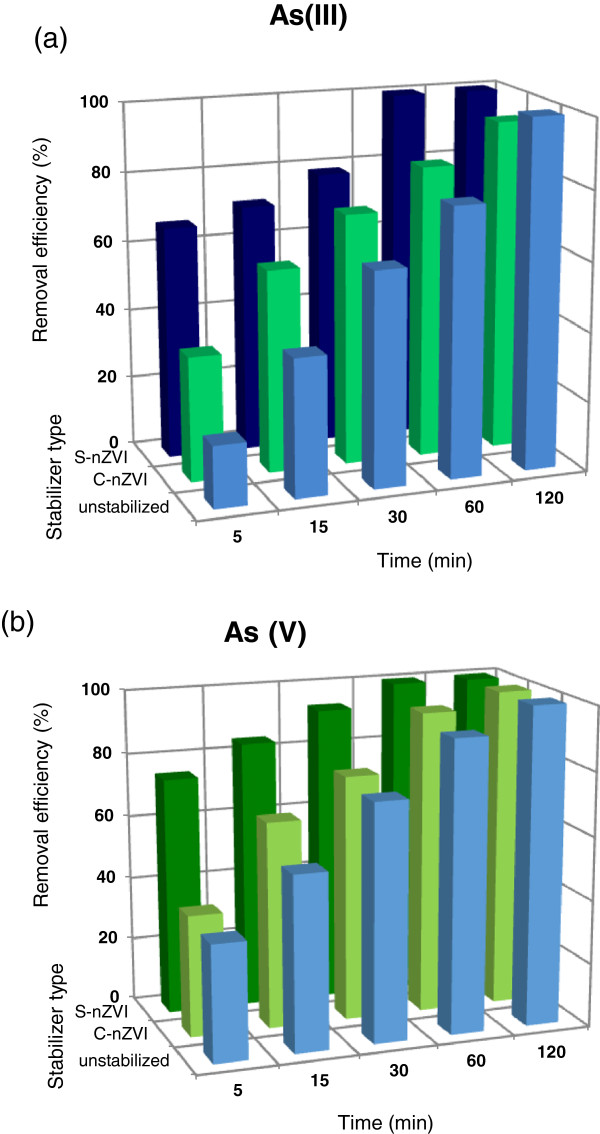
**Effect of different forms of Fe**^**0 **^**nanoparticles on the (a) As (III) and (b) As (V) removal.** [As] = 2 mg/L, [Fe]_0_ = 0.3 g/L and, pH _initial_ = 7 ± 0.1.

Figure 3 shows the results of batch experiments conducted for *comparative evaluation* of synthesized materials potential for As (III) and As (V) uptake from aqueous solution respectively. It was observed that stabilized ZVI nanoparticles exhibited greater reactivity and could yield higher removal capacity of both As (III) and As (V) in comparison with non-stabilized nanoparticles. Polymer surface coatings inhibited nZVI aggregation and enhanced their reactivity.

As can be seen S-nZVI samples removed both arsenate and arsenite with higher efficiency and faster rate than that of bare and CMC-stabilized nanoparticles; therefore this form of nanoparticles selected for using in remaining experiments. During the first 5 min of the reaction, 65.4% of As (III) and 74.6% of As (V) was removed in the presence of starch stabilized particles while only 17.7% and 35.6% of As (III) was removed using bare and CMC-stabilized nanoparticles respectively. In the case of As (V), bare and CMC-coated nanoparticles showed removal efficiency of about 36.6 and 38.2% at the first 5 min of the reaction respectively.

#### Arsenic removal mechanism using stabilized nanoparticles

Carboxcymethyl cellulose is a weak anionic polysaccharide and starch is a natural uncharged polymer. The use of CMC as a stabilizer can accelerate nucleation of Fe atoms during the formation of ZVI nanoparticles and, subsequently, forms a bulky and negatively charged layer via sorption of CMC molecules on the ZVI nanoparticles [26,39] and also according to Lin et al. [40] study, CMC was bound to particle surfaces in the form of bidentate bridging via the carboxylic group, which could provide both electrostatic and steric repulsion to prevent particle aggregation.

Herein, SEM images show, CMC kept nanoparticles physically separated and led to produce more stable nanoparticles (Figure 2c).

A fraction of the particles cannot be stabilized by any modifier and rapidly agglomerates to micron sized aggregates, as is also observed for unmodified NZVI. This nondispersible fraction is attributed to strong magnetic attractions among the larger particles present in the polydispersed NZVI slurry as the magnetic attractive forces increase [39].

Starch-stabilized particles are not all separated (referring to SEM image in Figure 2).

Nonetheless based on the aforementioned facts, starch stabilized nanoparticles had superior removal ability toward arsenic and displayed ~ 36.5% greater removal for As (V) and 30% for As (III) in comparison with C-nZVI.

The presumable explanation for this phenomenon is attribute to predominant removal mechanisms for arsenic remediation by NZVI which appear to be adsorption and/or surface precipitation, followed by redox reaction [41,42]. Coating the nanoparticles with the stabilizers greatly alters the surface potential, which can also affects sorption of the arsenic species [43]. The starch is a neutral polymer and stabilizes nanoparticles through steric repulsion arising from the osmotic force when the starch layers overlap as the particles collide. In fact, starch bridging prevents the nanoparticles from intense aggregating, and thus, maintains their high sorption capacity [19].

Since the predominant forms of As in natural ground-and surface waters (neutral pH like our experimental pH conditions) are arsenate (As (V), as oxyanions $H_{2}AsO_{4}^{-}$ and $HAsO_{4}^{2-}$) and arsenite (As (III), as the neutral $H_{3}AsO_{3}^{0}$ species) it might be postulated that the negatively charged layer on C-nZVI particles surface due to electrostatics repulsion do not favor the adsorbing arsenic oxyanions and thus the removal efficiency is reduced while starch as a surface buffer is reduced the effect of H^+^/OH^−^ on the surface charge of S-nZVI particles and therefore this type of stabilized Fe^0^ nanoparticles effectively removed arsenic from samples.

Similar result was confirmed in the An and Zhao [43] study in the arsenic immobilization using polysaccharide stabilized Fe-Mn nanoparticles. They also found that from the particle stabilization viewpoint, CMC is likely to be a more effective stabilizer than starch.

According to the results of the first stage of the present study, S-nZVI proved to be an outstanding material from arsenic removal standpoint, however there is a clear trade off to choosing the best stabilized nanoparticles form.

It should be noted that comparison of arsenic removal efficiency by some other nanomaterials shows that starch stabilized nanoparticles are more effective than the others.

Gupta et al. [44] reported that with an initial dose rate of 0.5 g/L of Chitosan zerovalent iron nanoparticles, concentrations of As (III) and As (V) were reduced from 2 mg/L to <5 μg/L, in less than 180 min, while in this study, almost complete removal of As (III) and As (V) (2 mg/L initial concentration) was achieved in less than 120 min when the starch zero-valent iron nanoparticles mass concentration was 0.3 g/L.

Shipley et al. [45] used magnetite nanoparticles for the removal of As and reported that magnetite nanoparticles at 0.5 g/L adsorbed 92.6 μg/L arsenate and 93.9 μg/L arsenite from an initial concentration of 100 μg/L in 1 h. Based on our observations in the next experimental phase, with material loading at 0.3 g/L and pH = 7, all the arsenic contamination (up to 500 μg/L) could be removed and the arsenic residual concentration could drop to zero in just 5 min.

After treatment in the conditions mentioned above, the soluble iron concentration that determines the amounts of iron ion leaching from S-nZVI was analyzed in filtrate supernatant fluid which was below the admissible limit set by the Institute of Standard and Industrial Research of Iran (0.3 mg/L). Nonetheless the iron concentration may be influenced by operating conditions such as pH, dissolved oxygen concentration and the presence of arsenic species.

### Kinetic study

**Figure 4:**
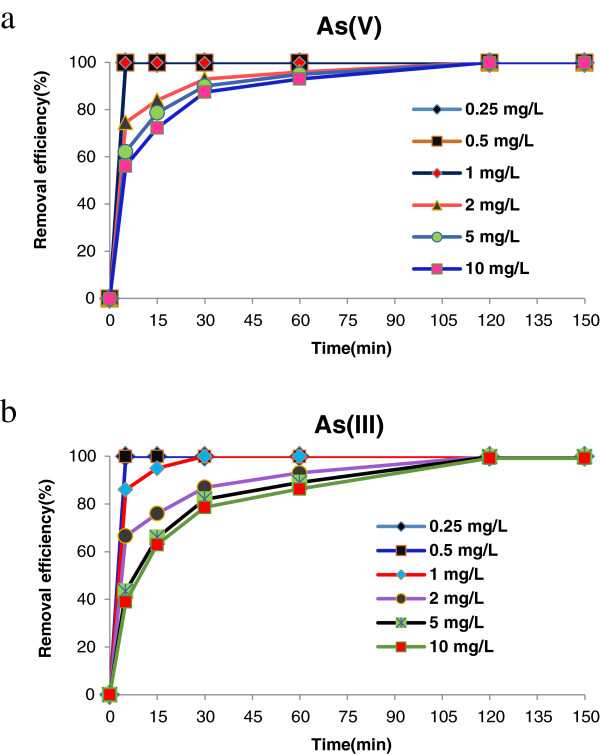
**Adsorption kinetics of As (V), (III) by S-nZVI (a, b).** Adsorbent dosage = 0.3 g/L, pH = 7.

Adsorption process on S-nZVI surface was very rapid and the time required to reach sorption equilibrium was 2 h for all concentrations of arsenic. Figure 4 shows the adsorption percentage of As (V) and As (III) on the Starched Fe^0^ nanoparticles.

The experimental data best fitted the pseudo-second-order kinetic model and the adsorption process might be chemisorptions which is suitable for sorption at low initial concentrations [46].

The linear equation of the pseudo-second-order model can be expressed as:

(3) $$\frac{t}{q_{t}}=\frac{1}{k_{2}{q_{e}}^{2}}+\frac{t}{q_{e}}$$

Where q_e_ and q_t_ (mg/g) are the amount of adsorbed arsenic at equilibrium and at time t (min), respectively. The results are presented in Table 1 however the adsorption data for As (III) and As (V) at an initial concentrations of 0.25, 0.5, 1 mg/L were not used in the fitting because all arsenic species were removed and the q_e_ value could not be determined.

**Table 1:** Kinetic model parameters of pseudo second-order

	**Parameters**			
**R**^**2**^	**k(g/(mg.min))**	**q**_**e**_**(mg/g)**	**Initial Cons.(mg/L)**	**Arsenic species**
0.9992	0.089	6.75	2	
0.9994	0.03	16.94	5	As(V)
0.9977	0.0077	34.12	10	
0.9985	0.024	6.66	2	
0.9986	0.0074	17.8	5	As(III)
0.9996	0.0055	31	10	

### Adsorption isotherms

The Langmuir and Freundlich equations are common isotherm models for single-solute adsorption which were tested with equilibrium data. The linear forms of the two models are:

(4) $$\mathrm{Langmuir}:\frac{C_{e}}{q_{e}}=\frac{C_{e}}{q_{\text{max}}}+\frac{1}{q_{\text{max}}\;K_{L}}$$

(5) $$\mathrm{Freundlich}:{\mathrm{logq}}_{e}={\mathrm{logK}}_{F}+\frac{1}{n}\log C_{e}$$

Where, q_e_ (mg/g) and C_e_ (mg/L) are equilibrium adsorption capacity and equilibrium arsenic concentration on the adsorbent and in the solution, respectively, q_max_ (mg/g) is the monolayer adsorption capacity; K_L_ (L/mg) is Langmuir adsorption constant related to the free energy of adsorption. K_F_ (mg/g)(mg/L)^-1/n^ and n (dimensionless) are constants related to the adsorption capacity and affinity, respectively. Calculated isotherm parameters related to the models using linear regression analysis for As (III) and As (V) adsorption are shown in Table 2.

**Table 2:** Adsorption isotherm parameters for arsenic removal by S-nZVI

	**Freundlich model**				**Langmuir model**		
**R**^**2**^	**n**	**K**_**F**_**(mg/g)(mg/L)**^**-1/n**^	**R**_**L**_	**R**^**2**^	**K**_**L**_**(L/mg)**	**q**_**m**_**(mg/g)**	**Arsenic species**
0.9575	2.17	14.36	0.06	0.9927	7.6	14	As(V)
0.9487	1.74	9.36	0.16	0.9935	2.48	12.21	As(III)

(RL > 1): unfavorable adsorption, (0 < RL > 1): favorable, (RL = 0): irreversible and linear adsorption (RL = 1).

The results confirmed that the Langmuir isotherm model is the highest fitted model for the adsorption process of both As (III) (q_max_ = 14 mg/g), As (V) (q_max_ = 12.2 mg/g). This implies that arsenic adsorption on S-nZVI is monolayer. Furthermore, the values of R_L_ for the Langmuir isotherm were between 0 and 1, and the Freundlich constant 1/n was smaller than 1 (As(V) 0.46 and As(III) 0.57), indicating a favorable process. The fundamental properties of the Langmuir isotherm can be explained in terms of dimensionless separation factor R_L_:(R_L_ = 1/(1 + k_L_.C_0_)).

### Effect of initial arsenic concentration and nanoparticles dose

**Figure 5:**
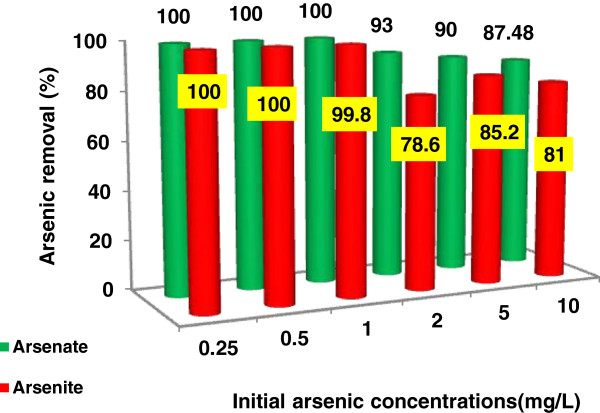
**The effect of initial arsenic concentration on the removal efficiency using S-nZVI, [Fe]**_**0**_ **= 0.3 g/L, reaction time =30 min, pH **_**initial**_ **= 7 ± 0.1.**

The laboratory results on the effects of initial arsenic concentration in removing arsenic from water are indicated in Figure 5.

Arsenic species could be removed using S-nZVI in a short time. for example, when the initial concentration was less than 1 mg/L, the removal fraction of both arsenite and arsenate was more than 99% and there was very little arsenic left in the solution after only 30 min treatment however with increasing initial concentration of contaminants, the removal efficiency decreases. It is clear that for lower initial concentrations of arsenic, adsorption was very fast.

For high arsenite concentration of 10 mg/L, the residual concentration in the solution was reduced to about 1.4 mg/L after 60 min (Figure 4b). When initial concentration increase, only a fewer active sites for adsorption of arsenic remain on NZVI and the removal percentage is reduced.

This result has been confirmed by Khodabakhshi et al. [47] which reported As (III) removal with magnetite nanoparticles is inversely related to initial arsenic concentrations.

**Figure 6:**
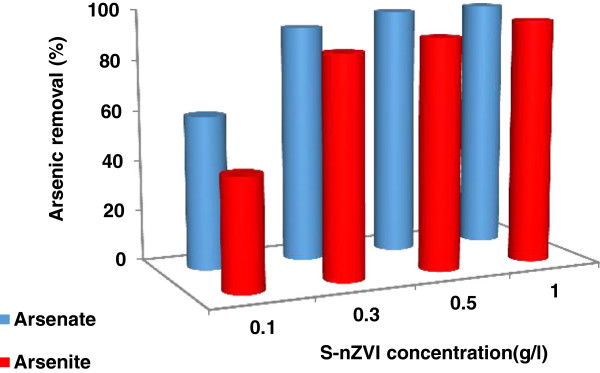
**The effect of S-nZVI concentration on the As (III) and As (V) removal from aqueous solution, [As] = 2 mg/L, reaction time =30 min, pH **_**initial**_ **= 7 ± 0.1.**

Figure 6 demonstrates the variation in As (III), (V) removal in water samples as a function of S-nZVI concentrations in solution.

It is clear, the percentage removal of As (V) is more than As (III) over the range of nano particle concentrations and the removal of both arsenite and arsenate increased from ~ 44% and ~ 60% to more than 95% when the dose of S-nZVI applied increased from 0.1 to 1 g/L over a 30 min period. This is accordance with the fact that the adsorptive and active sites available for a fixed concentration of arsenic on the nanoparticles surface increased when the nanoparticles loading increased.

A similar dose dependency trend in arsenic removal using magnetite [45], goethite [48] and ultrafine iron oxide (α-Fe_2_O_3_) nanoparticles [49] has been reported in the literature.

### Effect of pH

**Figure 7:**
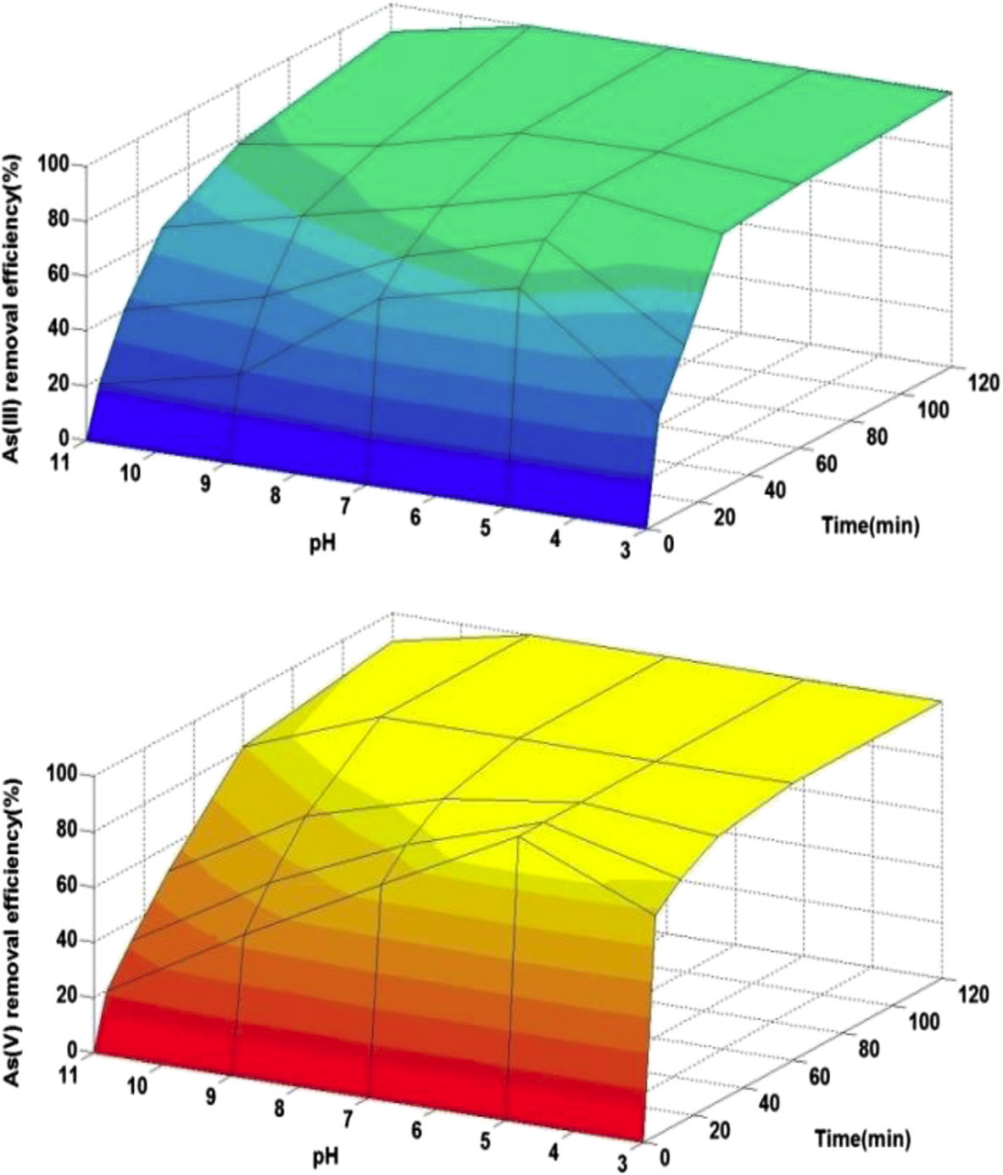
**Effect of pH on the As (III) and As (V) removal efficiency using S-nZVI, [As] = 2 mg/L, [Fe]**_**0**_ **= 0.3 g/L.**

The percentage removal of arsenic species using starch stabilized Fe^0^ nanoparticles as a function of pH is separately presented in Figure 7.

The removal of both As (III) and As (V) species was found to be optimum at pH range of 5–7 and displayed a maximum uptake at around pH 5 with the amount of 77.26 and 100% respectively. The removal efficiency of arsenite was declined to approximately 30% and 18% and the arsenate removal to approximately 48% and 20% in solution during the first 5 min of the reaction, by changing pH to 9 and 11 respectively.

This phenomenon can be elucidated from speciation of arsenic in solution. The dissociation constants of aqueous As(III) are pK_a1_ = 9.17, pK_a2_ = 12.1 and pK_a3_ = 13.4. When the pH is above 9.17, anionic $H_{2}AsO_{3}^{-}$ is mainly As(III) species and the predominant species at pH below 9.17 is neutral, namely H_3_AsO_3_. As(V) predominantly exists as $H_{2}AsO_{4}^{-}$ and $HAsO_{4}^{2-}$ in the pH range of 2.2 to 11.5 [50,51].

On the other hand, removal of arsenic occurs, predominantly by adsorption on iron oxide species generated by corrosion of zerovalent iron. At pH > 8, these iron oxides present in the monomeric anionic form $\text{Fe}{\left(HO\right)}_{4}^{-}$[52] and repel negative charge arsenic species. As a result, the removal of both arsenic species was significantly reduced due to electrostatic repulsion and also competition between OH^−^ and arsenic oxyanions for the active sites on the surface of nanoparticles as well at pH above 9.

Ferrous iron (Fe^2+^) is a primary product from metallic iron corrosion process [53] which according to Taha and Ibrahim pH and Fe^2+^ has a very strong reverse correlation in NZVI system [54] and in low pH iron hydroxides are present as cationic monomers of FeOH^+2^ and $\text{Fe}{\left(OH\right)}_{2}^{+}$[47], hence arsenic adsorption should be enhanced in acid conditions. Interestingly, our results indicated around pH 3 the arsenic sequestration rate was lower than the maximum rate. Probably the diminished arsenic uptake at pH below 5 can be attributed to the break-down smaller segments of starch and elevated solution viscosity and also decomposition of Fe^0^ nanoparticles at pH < 5. The study carried out by An et al. [19] shows similar results and they reported that 13% of starch-bridged magnetite nanoparticles at pH 3.2 was dissolved.

It is evident that S-nZVI is effective in slightly acidic and natural pH values. Whereas most natural waters are at near neutral conditions, this result is very desirable.

### Comparative evaluation of arsenic removal by different adsorbents

At final stage of the study, the S-nZVI adsorption capacity (q_max_) has been compared with some reported data for different adsorbents (Table 3). It is evident that Starched Fe^0^ nanoparticles are relatively good adsorbent and have superior performance in removing both As(III) and As(V) from aqueous solutions in a short time. Hence, they should be subsequently confirmed on real contaminated water bodies. Future research could focus on the effect of various diverse ions/competing co-ions upon adsorption of arsenic and the reusability of nanoparticles.

**Table 3:** Comparison of adsorption capacity for arsenic with various nanoadsorbents

			**Experimental conditions**		
**Adsorben**	**Ads. capacity (mg/g)**	**pH**	**Concentrations (mg/L)**	**Arsenic species**	**Reference**
Fe_2_O_3_	4.6	7	1-4	As(V)	[55]
Fe_3_O_4_	0.2	9	0.1-2	As(V)	[56]
α-FeOOH	76.3	3	5-500	As(V)	[48]
Fe_3_O_4_-γFe_2_O_3_	4.85	6.5	2	As(V)	[57]
	4.75	6.5	2	As(III)	
Crystalline TiO_2_	37.5	7	-	As(V)	[58]
CuO nanoparticles	1.068	7	0.5-1	As(III)	[59]
Chitosan nZVI	119	7	1-60	As(V)	[58]
	94	7	1-60	As(III)	[59]
Amorphous ZrO_2_ nanoparticles	32.4	7	1-60	As(V)	[44]
	83	7	0.3-100	As(III)	[46]

It should be noted that Nano zero-valent iron is the cheapest among other nanomaterials such as Nano TiO_2_, Nano-Magnetite and Nano Iron-Oxide [60], however depending on the type and amount ordered nZVI, it costs in the range of £50-150 per kg. In order to compete against existing treatment methods, the price of nZVI must reduce to approximately < £10 per kg [53]. In spite of this barrier, their use is likely to increase at the point-of-use/entry scale.

## Conclusion

In this work, starch was proven to be an effective stabilizer for Fe^0^ nanoparticles. The starch stabilized nanoparticles demonstrated comparable high removal efficiency towards arsenic species without the need of pre-oxidation and/or pH adjustment.

As a common trend, it was observed that an increase of S-nZVI loading and contact time and a decrease of pH and initial arsenic concentration determined a higher efficiency of arsenic removal. The optimum removal efficiency of both arsenite and arsenate was found at pH range of 5–7.

As (V) removal was faster than As (III) and both species removal increased with S-nZVI mass (0.1-1 g/L) attaining more than 95% after 30 min of time contact with the 0.3 g/L nanoparticles concentration.

Overall, starch as a low-cost and green stabilizer could be a suitable candidate to enhance iron nanoparticles reactivity for the removal of arsenic contamination from aqueous solutions.

## Competing interests

The authors declare that they have no competing interests.

## Authors’ contributions

This study is a part of MSc. approved thesis. The study was directed by MM who is the first author and supervised all the experiments and edited the manuscript. SN (Corresponding author) performed all the experiments and drafted the manuscript. AKh advised the experimental methods. SN read and approved the final manuscript. ASH was involved in discussion of the results. All authors have read and approved the final manuscript.
